# Diet, Microbiota, Obesity, and NAFLD: A Dangerous Quartet

**DOI:** 10.3390/ijms17040481

**Published:** 2016-04-01

**Authors:** Mariana Verdelho Machado, Helena Cortez-Pinto

**Affiliations:** 1 Departamento de Gastrenterologia, Hospital de Santa Maria, Centro Hospitalar Lisboa Norte (CHLN), 1649-035 Lisbon, Portugal; mverdelhomachado@gmail.com; 2 Laboratório de Nutrição, Faculdade de Medicina de Lisboa, Universidade de Lisboa, Alameda da Universidade, 1649-004 Lisboa, Portugal

**Keywords:** nonalcoholic fatty liver disease, microbiota, diet, obesity, dysbiota, probiotics

## Abstract

Recently, the importance of the gut-liver-adipose tissue axis has become evident. Nonalcoholic fatty liver disease (NAFLD) is the hepatic disease of a systemic metabolic disorder that radiates from energy-surplus induced adiposopathy. The gut microbiota has tremendous influences in our whole-body metabolism, and is crucial for our well-being and health. Microorganisms precede humans in more than 400 million years and our guest flora evolved with us in order to help us face aggressor microorganisms, to help us maximize the energy that can be extracted from nutrients, and to produce essential nutrients/vitamins that we are not equipped to produce. However, our gut microbiota can be disturbed, dysbiota, and become itself a source of stress and injury. Dysbiota may adversely impact metabolism and immune responses favoring obesity and obesity-related disorders such as insulin resistance/diabetes mellitus and NAFLD. In this review, we will summarize the latest evidence of the role of microbiota/dysbiota in diet-induced obesity and NAFLD, as well as the potential therapeutic role of targeting the microbiota in this set.

## 1. Introduction

Nonalcoholic fatty liver disease (NAFLD) refers to the ectopic accumulation of fat in the liver. In its primary form, NAFLD is the hepatic manifestation of metabolic dysfunction associated with energy surplus-induced adiposopathy. The term adiposopathy has only recently been introduced in the medical lexicon and translates the adipose tissue dysfunction that occurs, in susceptible individuals, as a consequence of chronic positive caloric balance and sedentary lifestyle [1]. The true significance of hepatic steatosis as a contributing player in obesity-induced dysmetabolism and global metabolic and cardiovascular health is still unclear [2]. Regarding liver health, although most patients will present stable, non-progressive disease, the high prevalence of this condition explains why NAFLD is the number one cause of chronic liver disease in Western world and will predictably be the number one cause of end-stage liver disease in the near future [3].

Little more than a decade ago, a major breakthrough linked the gut microbiota to the pathogenesis of obesity and NAFLD [4]. Since then, medical research in the field has flourished exponentially. However, huge gaps in knowledge still preclude us to have effective therapeutic strategies for obesity and NAFLD that act through modulation of gut microbiota.

The gut microbiota comprises 10 to 100 trillion microbes. The gut microbiota is composed by bacteria, archea, virus, and fungi, being dominated by four main phyla of bacteria: Firmicutes, Bacterioidetes, Actinobacteria, and Proteobacteria, which represent more than 95% [5,6]. The collective genome of the gut microbiota, referred to as a microbiome, contains at least 100 times more genes than the human genome [6]. Those extra genes are crucial to maintain our homeostasis. In fact, the gut microbiome is enriched in several genes important for glycans and aminoacids metabolism, xenobiotics metabolism, methanogenesis, and biosynthesis of vitamins [6]. This explains why the gut microbiota contributes to host nutrition, bone mineral density, modulation of the immune system, xenobiotics metabolism, intestinal cell development and proliferation, and protection against pathogens [7].

One important question still not fully answered is if there is a core microbiota common to humans. In fact, although culture-based studies suggest that healthy humans would share the same gut bacterial species, culture-independent studies showed that each individual harbors a unique collection of bacterial strains and species [7,8]. Not only gut microbiota is specific to individual, it is also highly resilient, promptly returning to baseline after perturbation [7,9,10,11]. However, recovery may be impaired with recurrent perturbation [12]. Interestingly, despite the unique individual gut microbiota, humans share similar functional gene profiles, implying a core functional microbiome [8].

The composition of the gut microbiota is regulated by (a) external factors such as vaginal *versus* cesarean section delivery, breast feeding, antibiotics, pre/probiotics, diet, hygienic habits, and random chance resulting in a colonization cascade; (b) interaction with the host such as genetics, Paneth cell function, mucus composition, secretion of antimicrobial peptides; and (c) interaction between microbes, which can result in competition or cooperation [5,13,14].

In this review, we will summarize the latest research on the interplay between diet, gut microbiota, obesity, and fatty liver disease. We will also discuss the evidence of microbiota-targeting approaches in the treatment of NAFLD.

## 2. Microbiota and Obesity

The first clue on the role of the microbiota in the pathogenesis of obesity came from Backhed *et al.* [4] studies. They compared body weight gain in germ free mice and conventionally raised mice, and found that the latter gained more weight, with increased adipose tissue and body fat percentage, which could not be explained by different diet intake. Importantly, metabolic status was worse in conventionally raised mice, with higher leptin levels, lower insulin sensitivity and greater fat accumulation in the liver. Further supporting the concept that body weight was regulated by gut microbiota, transplantation of microbiota harvested from conventionally raised mice into germ free mice resulted in an increase in body weight and decrease in insulin sensitivity [4]. Moreover, the same group showed that, not only germ-free mice were leaner than conventionally raised mice, they were also resistant to western-type high-fat diet induced obesity [15]. Lastly, studies on animal models showed us that not all microbiota has the same effect on metabolism, and raised the possibility of an obesity-specific microbiota. In fact, transplantation of microbiota harvested by either genetically-obese ob/ob mice [16] or high-fat diet induced obese mice [17] into germ free mice mimicked the obese insulin resistant phenotype. Supporting the animal data, a small human study in male patients with the metabolic syndrome submitted to autologous or allogenic (from a lean donor) intestinal microbiota via duodenal tube, showed improvement in insulin sensitivity when the donor was lean [18].

Since then, several groups tried to characterize the obese-associated microbiota. Studies in either genetically or diet-induced obese mice showed differences in the microbiota when comparing with lean mice. Obese mice consistently showed a decrease in Bacterioidetes and an increase in Firmicutes (particularly from the class Millicutes) [19,20,21]. This increase in Firmicutes associated with an increase in enzymes able to breakdown indigestible polysaccharides from diet and producing short chain fatty acids (SCFA) [19]. Obese mice also presented an increase in methanogenic Archea, which associates with a lower hydrogen partial pressure and optimization of bacterial fermentation [19].

Studies in human obesity showed lower microbial diversity and similar differences in the intestinal microbiota as suggested by animal studies [22,23,24].

In summary, there is an obesity-associated gut microbiota, and obesity can be infectiously transmitted by transplant of that microbiome, suggesting that it is the microbiota itself that promotes obesity. Supporting this concept, a prospective study in children showed that the risk of being overweight at seven years old could be predicted by the composition of gut microbiota at six months old, which associated with lower prevalence of *Bifidobacterium* and higher of *Staphylococcus aureus* [25].

Obese mice waste less energy in the stools as compared to lean mice, and as little as a 20% decrease in fecal Bacterioidetes associates with 150 Kcal decrease in energy harvest from the diet [26]. The microbiota can modulate body weight through several mechanisms. One such mechanism is the differential fermentation of indigestible carbohydrates in SCFA: butyrate, propionate, and acetate [27]. Overall, colonic-derived SCFA account for 10% of harvested energy from the diet, with acetate being the main source of energy [28]. Butyrate and propionate are considered anti-obesogenic, and acetate mainly obesogenic. Interestingly, while acetate and propionate are mainly produced by the phylum Bacterioidetes, butyrate is mainly produced by Firmicutes (the most important belonging to clostridial lusters IV and XIVa: *Faecalibacterium prausnitzii*, *Eubacterium rectale*, and *Rosuberia intestinalis*) [29,30]. Butyrate is a major source of energy for colonocytes, increasing intestinal health and potentially decreasing gut permeability and preventing metabolic endotoxemia [31]. Butyrate also seems to positively affect insulin sensitivity through stimulation of the release of the incretins glucagon-like peptide-1 (GLP-1) and gastric inhibitory polypeptide (GIP) [32]. Both butyrate and propionate can increase the expression of the anorexigenic adipokine leptin [33]. Other beneficial effects of propionate are inhibition of resistin expression by the adipose tissue [34] and inhibition of cholesterol synthesis through inhibition of acetyl-CoA synthetase and via buffering fatty acids to gluconeogenesis in detriment of cholesterol synthesis [27]. On the other hand, acetate is the most substantially absorbed SCFA, and is a substrate for lipogenesis and cholesterol synthesis in the liver and adipose tissue [27]. Finally, SCFA bind to specific receptors in the gut, liver, and adipose tissue, GRP43 and GRP41, which seem to have anti-inflammatory and metabolic actions that protect from obesity [28]. Interestingly, supplementation of oral butyrate in mice fed a Western diet, partially prevented liver steatosis and inflammation, while having no effect on obesity [35].

Gut microbiota can also decrease the intestinal expression of the adipose tissue lipoprotein lipase inhibitor fasting induced adipose factor (Fiaf), also known as angiopoietin-like factor IV (ANGPTL4). The net result is increased uptake of fatty acids in the adipose tissue and liver, favoring expansion of the adipose tissue and hepatic steatosis. Microbiota also prevents the beneficial action of Fiaf in the expression of peroxisome proliferator-activated receptor (PPAR)-1α coactivator (PGC) and fatty acids oxidation [15,36]. Other mechanisms by which gut microbiota promote obesity are an increase in mucosal gut blood flow enhancing nutrients absorption [37]; inhibition of adenosine monophosphate-activated protein kinase AMPK in the liver and muscle, and consequently inhibiting peripheral fatty acids oxidation and insulin resistance [15]; and modulation of the pattern of conjugated bile acids and its function in lipid absorption [38].

Obesity itself may also change the microbiota, independently of the diet. For example, leptin, an adipokine whose levels are increased in obesity, has a direct role regulating the gut microbiota composition, through the modulation of antimicrobial peptides secretion by Paneth cells in the gut [39]. As such, a vicious circle between microbiota and adiposity promotes further worsening of obesity.

## 3. Microbiota and Nonalcoholic Fatty Liver Disease (NAFLD)/Nonalcoholic Steatohepatitis (NASH)

NAFLD strongly associates with obesity. The aggregate data suggests that the gut microbiota may play a significant role in the pathogenesis of obesity, as such it would be logical to think that the gut microbiota also plays a role in the development of NAFLD and its progressive form, nonalcoholic steatohepatitis (NASH). Indeed, that seems to be the case. Transplanting harvested microbiota from conventionally raised mice to germ free mice, besides increasing body weight, it also increases the fat content in the liver [4]. Furthermore, treatment with antibiotics protected from hepatic steatosis in different dietary and genetic obese rodent models [40,41]. However, the association between gut microbiota and NAFLD goes beyond the association with obesity.

Several studies in animal models and patients with NAFLD or NASH showed an association with small bowel overgrowth and increased intestinal permeability [42,43,44,45,46,47,48,49]. Brun *et al.* [45], compared two strains of genetically obese mice, leptin deficient ob/ob and leptin-resistant db/db, with lean control mice. They found that obese mice had increased intestinal permeability with lower intestinal resistance and profound changes in the cytoskeleton of cells in the intestinal mucosa. In association with increased gut permeability, obese mice, as compared to lean mice, had higher circulating levels of inflammatory cytokines and portal endotoxemia. Finally, hepatic stellate cells from obese mice expressed higher levels of the lipopolysaccharide (LPS) co-receptor CD14, and responded with a more inflammatory and fibrogenic phenotype after stimulation with LPS [45]. Furthermore, a study compared NAFLD patients with healthy subjects, and found that patients with NAFLD had an increased susceptibility to develop increased intestinal permeability after a minor challenge with low dose aspirin [46]. Concordant with those observations, obesity and NAFLD associates with metabolic endotoxemia, *i.e.*, increased blood levels of lipopolysaccharide (LPS), a component of the wall of Gram-negative bacteria that binds to specific receptors, toll like receptor-4 (TLR-4), and can promote hepatic and systemic inflammation [47,49,50]. Verdam *et al.* [51] also showed an increase in plasma antibodies against LPS in patients with NASH as compared to healthy controls, which progressively increased with increased severity of liver disease. The role of LPS is highlighted by the study by Cani *et al.* [50] in which LPS injections in mice simulated the effects of a high-fat diet, in terms of weight gain, insulin resistance, and development of NAFLD. Furthermore, mice deficient in TLR-4 are not only protected from LPS-induced obesity and NAFLD, but also from high-fat diet-induced obesity and NAFLD [50], as well as NAFLD and NASH in different rodent models [47,52,53,54].

Perturbations in the metabolism of bile acids seem to have a prominent role in the pathogenesis of NAFLD [55]. Bile acids are not only critical in the absorption of fat, they are also signaling molecules with actions in their own metabolism, as well as energy, lipoproteins, and glucose metabolism, through its receptors farsenoid X receptor FXR and TGR5. There is a known mutual influence between bile acids and gut microbiota. Bile acids have potent antimicrobial properties [56]. On the other hand, the gut microbiota increases the diversity of bile acids through the deconjugation, dehydrogenation, and dehydroxylation of primary bile acids. In fact, conventionally raised mice, as compared to germ free mice presented a decrease in tauro-conjugates (which are potent FXR antagonists and hence positive regulators of bile acids synthesis), while maintaining levels of the more toxic cholic acid [57].

Recently, two studies elegantly demonstrated that NAFLD could be a transmissible disease, through gut microbiota. Le Roy *et al.* [58] fed mice with high fat diet for 16 weeks, and while most of the animals developed NAFLD, insulin resistance, and systemic inflammation (dubbed responders), some mice did not develop NAFLD or insulin resistance (dubbed non-responders). When they transplanted germ free mice with microbiota harvested from those animals, they obtained a metabolic and liver phenotype only if the donors were responders. Furthermore, mice with a genetic deficiency of the inflammasome in the gut exhibited a perturbed gut-innate immunity and an abnormal gut microbiota with increased Bacterioidetes (particularly from the family Porphyromondaceae) and decreased Firmicutes. Those mice developed worse liver damage when fed NASH-inducing diets, with increased steatosis, inflammation, and aminotransferases levels, as compared to their wild type counterparts. Interestingly, co-housing those transgenic mice with wild type mice turned the latter more sensitive to the diet-inducing NASH, effect that was abrogated by concomitant treatment with antibiotics [59]. Lastly, de Minicis *et al.* [60] modulated gut microbiota through high-fat diet (which induced an increase in Proteobacteria), before submitting mice to bile duct ligation. Those mice developed worse fibrosis than chow diet fed mice. They simulated the increased susceptibility to fibrosis by transplanting gut microbiota from high-fat diet fed mice, which was even worse when they selectively transplanted Gram-negative bacteria.

The gut microbiota also seems to have a role in NAFLD-associated hepatocarcinogenesis. Yoshimoto *et al.* [61] showed that, in different animal models of obesity, dysbiota associates with increased deoxycholic acid reaching the liver through the enterohepatic circulation. This bile acid was able to produce a senescence phenotype in hepatic stellate cells that induced a secretory profile able to promote inflammation and tumorigenesis.

Several studies in adult patients, have tried to evaluate if the presence of NAFLD associates with a specific dysbiota [62,63,64,65,66,67] (Table 1). Those are small studies, with different populations and controls and often without histological diagnosis. Furthermore, statistical significance was achieved in different categories in the taxonomic hierarchy. Though NAFLD/NASH seems to share some of the microbiota specificities associated with obesity, at the phylum level, only one study found NASH to be associated with a decreased percentage of Bacterioidetes [63]. The two studies that compared patients with NAFLD with healthy controls found an increase of the genus *Lactobacillus*, and a decrease in the family Ruminococcaceae in NAFLD patients [64,67]. Regarding the association with *Lactobacillus*, it is surprising, since several species from this genus are frequently used as probiotics. *Lactobacillus* are lactic acid bacteria that can inhibit pathogens, enhance the epithelial barrier function, and modulate immune responses [68], actions that would seem protective in the pathogenesis of NAFLD/NASH. However, *Lactobacillus* may associate with the production of volatile organic compounds such as acetate and ethanol [69], which may be important in the pathogenesis of obesity and NAFLD [64]. In fact, the genus *Lactobacillus* comprises over 180 species and a wide variety of organisms; while some can only produce lactic acid from the fermentation of sugars (e.g., *L. acidophilus* and *L. salivarius*), other can also produce ethanol (e.g., *L. casei*, *L. brevis* and *L. plantarum*). Again, the decrease of Ruminococcaceae may also translate to a decrease in the production of SCFA such as butyrate, since many bacteria from that family produce butyrate [70]. A decrease in butyrate-producing bacteria, such as the genus *Faecalibacterium* [70] has also been associated with NASH, as compared to healthy controls [65]. As compared to healthy subjects, patients with NAFLD also showed increased percentage of bacteria from the genera *Escherichia* and pathogenic *Streptococcus*, both known to potentially induce persistent inflammation in the intestinal mucosa, and to be associated with inflammatory bowel disease [71,72]. In accordance, patients with NAFLD exhibited higher expression of proinflammatory cytokines in the intestinal mucosa [67]. Some *Escherichia* species also produce ethanol, which can further increase gut permeability. In fact, children with NASH also displayed increased levels of *Escherichia* bacteria in their stools [73].

Spencer *et al.* [62] evaluated an interaction between choline metabolism and microbiota in the development of NAFLD. They studied 15 inpatient women and submitted them to depletion of choline. They found that differences in two classes of bacteria (decrease in Gammaproteobacteria and increase in Erysipelotrichi), in association with genetic polymorphisms in phosphatidylethanolamine *N*-methyltransferase (PEMT, a key enzyme in the choline metabolism), could predict the susceptibility to develop NAFLD with choline depletion. This is highly relevant, because the median choline intake in the United States is half the recommended dose (recommended dose: 550 mg per day) [74]. Gut microbiota can further promote choline depletion by hydrolyzing choline to trimethylamine, which can be further metabolized in the liver into the toxic compound trimethylamine N-oxide (TMAO). Interestingly, feeding mice with high fat diet is known to shift the gut microbiota into a choline degradation profile [75].

In patients with NAFLD, the presence of NASH associated with an increase in the genus *Bacteroides* [66]. This skew in favor of *Bacteroides* may translate to an increase in the toxic bile deoxycholic acid, which is known to induce apoptosis in hepatocytes and to be increased in patients with NASH [76,77]. Furthermore, *Bacteroides* has been associated with an increase in branched-chain fatty acids derived from aminoacids fermentation, which have diabetogenic potential [78]. Lastly, in patients with NAFLD, the presence of significant fibrosis also associated with increased content of the genus *Ruminococcus*, which is difficult to interpret, since it is a highly heterogeneous genus including both potentially beneficial and detrimental species [66]. Nevertheless, some species from the *Ruminococcus* genus are pro-inflammatory and able to produce ethanol [79,80,81], two potential pathogenic mechanisms in the progression of NAFLD.

NAFLD and particularly NASH also seem to associate with specific changes in the oral microbiota. Yoneda *et al.* [82] studied 150 patients with NAFLD (of those 102 with NASH) and 60 healthy controls, and found that infection with *Porphyromonas gingivalis* (the major cause of periodontitis) tripled the risk for NAFLD and quadrupled the risk for NASH, independent of ge and metabolic syndrome. In 10 patients with NAFLD, treatment of periodontitis prompted an improvement in liver enzymes [82]. Furthermore, in patients with NASH, positive immunohistochemistry for *P. gingivalis* associated with increased fibrosis [83]. In mice fed high-fat diet, infection with *P. gingivalis* associated with endotoxemia and increased blood levels of proinflammatory cytokines, as well as worse liver disease, including worse fibrosis [82,83].

**Figure 1:**
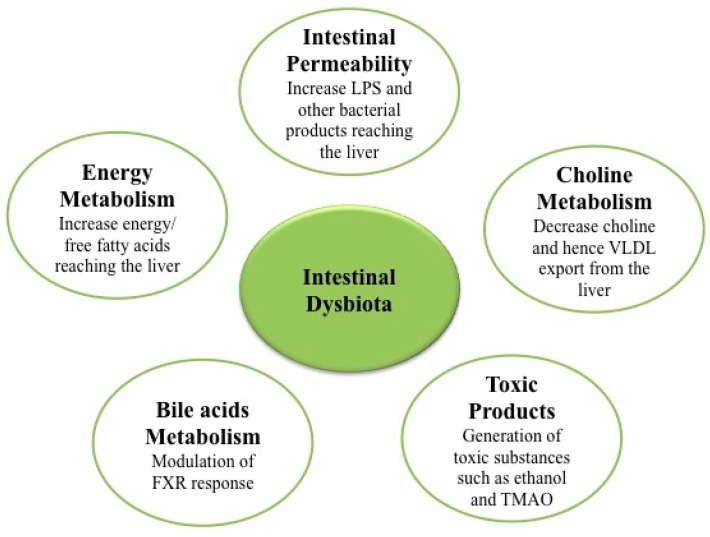
Nonalcoholic fatty liver disease (NAFLD) associated mechanisms of intestinal dysbiota.

In summary, gut microbiota can contribute to the development and progression of NAFLD via several mechanisms: (a) modulation of energy homeostasis and energy harvested from diet with associated obesity and dysmetabolism [4,26]; (b) modulation of intestinal permeability promoting endotoxemia as well as other microbe products that promote systemic and hepatic inflammation [50]; (c) modulation of the choline metabolism (required for very low density lipoproteins VLDL synthesis and export of lipids from the liver) [75]; (d) generation of endogenous ethanol as well as other toxic products such as TMAO [73,84,85,86]; and (e) modulation of bile acids homeostasis and FXR function [87,88] (Figure 1).

## 4. Diet and Microbiota

Both the quality and quantity of our diet strongly modulate the gut microbiota. Different diets associate with different compositions of the microbiota. De Fillipo *et al.*’s [89] work beautifully translates this concept. They compared the fecal microbiota of European children (who ate a modern Western diet) with children from a rural African village of Burkina Faso (which ate a high-fiber diet, similar to the ancient diet soon after the birth of agriculture). Children from Burkina Faso had a decreased Firmicutes/Bacterioidetes ratio, a higher percentage of bacteria from the genera *Prevotella* and *Xylanibacter* (known to be equipped with enzymes in the degradation of indigestible carbohydrates), and a decrease in the proinflammatory Enterobacteriaceae, *Shigella* and *Escherichia*. They also had higher amounts of SCFA in the stools. This study suggests that gut microbiota coevolved with the polysaccharide rich diet in order to maximize energy intake from fibers [89].

How quickly can a change in the diet induce differences in the microbiome? In mice, we can induce changes in the gut microbiome after just one single day on a different diet [17]. Studies in humans also showed diet-driven changes in the intestinal microbiota occurring as early as in three to four days [90]. In a clinical study, David *et al.* [91] were able to induce differences in microbiota, that would be metabolically more fit to the type of diet administered, entirely animal or entirely plant products, in just five days. Furthermore, volunteers placed on a three-day high or low-calorie diet, showed that even this short-term increase in energy intake, associated with an increased Firmicutes/Bacterioidetes ratio, correlated with a decrease in the proportion of energy loss in the stools [26]. Indeed, diets enriched in fibers associate with an increase in the fecal loss of energy [92]. However, after stopping the diet, microbiota quickly returned to the basal state, translating the high resilience of our gut flora. Similarly, a dietary intervention in obese or overweight subjects, consisting of administering an energy-restricted high protein diet during six weeks, increased the diversity of species in the gut, along with decreased adiposity, which reverted to basal levels after the diet was stopped [93]. In contrast, long-term diets were able to induce more profound changes in the microbiota than short-term ones [94].

Chronic high-fat diet feeding in mice is known to change gut microbiota with progressive increase in Firmicutes and decrease in Bacterioidetes [20,21]. One important question regarding diet-induced changes in the microbiota is whether it is the composition of the diet or the number of calories ingested that has an effect on gut flora. Also, is diet or obesity itself the important factor for our gut health? Several lines of evidence suggest that both quantity and quality of the diet modulate gut microbiota. Mice deficient in resistin-like molecule β are resistant to high-fat diet induced obesity, however they still shift their gut microbiota with a decrease in Bacterioidetes and increase in Firmicutes as well as Proteobacteria, in response to those diets, in a similar way as their wild type counterparts [95]. This suggests that it is diet and not obesity, the critical factor determining the gut microbiota. On the other hand, when genetically obese leptin resistant mice were pair-fed with wild type mice, they still maintained the same differences in gut microbiota as genetically obese leptin resistant mice fed *ad libitum* [39]. This suggests that leptin itself (and hence the obesity state) may modulate gut microbiota independently of the diet.

Suggesting a critical effect on the composition of the diet, different formulations of high-fat diet, with different percentages of saturated and polyunsaturated fatty acids, seem to have different effects on the gut microbiota. Feeding mice with diets with higher percentage of saturated fatty acids not only seemed to associate with worse weight gain and hepatic steatosis, it also induced more profound changes in the microbiome, with a decrease in diversity and an increase in the Firmicutes/Bacterioidetes ratio [96]. Concordant with the concept of diet composition and gut microbiota crosstalk, mice were fed with either low-fat diet for 35 weeks (remaining lean), high-fat diet for 35 weeks (becoming and remaining obese), low-fat diet for 12 weeks followed by restricted intake of low-fat diet for 23 weeks (to maintain a 20% reduction in body weight), or high-fat diet for 12 weeks followed by restricted intake of high-fat diet for 23 weeks (in order to gain weight and then maintain a 20% reduction in body weight) [97]. The authors found that, regardless of weight status, low-fat diets induced the higher abundance of Firmicutes due to two species from the genus *Allobaculum*, and the high-fat diets induced the higher abundance of non-*Allobaculum* Firmicutes, Bacterioidetes and Mucispirillum. The aggregate animal data suggest a contribution of the quality of the diet *versus* the caloric intake in the composition of the gut microbiota.

Similar conclusions regarding the importance of quality *versus* quantity of the diet, can be taken from a study on obese volunteers that ate one of two isocaloric diets: low carbohydrates/high fat or high carbohydrates/low fat [98]. While the former diet associated with a decrease in fecal SCFA and *Bifidobacterium*, the latter associated with an increase in total anaerobes in fecal samples.

Fava *et al.* [99] studied subjects at increased risk for the metabolic syndrome. Those subjects were given a high saturated fat diet for four weeks and, subsequently, randomized for one of the following diets: high saturated fat diet, high monosaturated fat (MUFA)/high glycemic index diet, high MUFA/low glycemic index diet, high carbohydrate/high glycemic index diet and high carbohydrate/low glycemic index diet. They found that high carbohydrate diets (low fat) increased fecal *Bifidobacterium* and improved glucose metabolism, however if the diet had high glycemic index, it associated with an increase in fecal Bacteroides (which were associated with NASH in patients with NAFLD [66]), and if the glycemic index were lower, it associated with an increase in *Faecalibacterium prausnitzii* (which seems beneficial in protecting from NASH [65]). Furthermore, high saturated fat diets associated with increased fecal SCFA content. In conclusion, the Fava *et al.* [99] study beautifully translates that different compositions of isocaloric diets can modulate the gut microbiota, with potential impact in the risk for the development of the metabolic syndrome and NASH.

Studies in mice showed that high-fat diets could increase fecal content of hydrogen sulfide producing bacteria such as from the family Desulfovibrionaceae. This is a relevant effect since hydrogen sulfide is toxic to colonocytes, perturbing intestinal barrier function and increasing endotoxemia [100]. Another important association was made with *Akkermansia muciniphila*, a specific type of mucin-degrading bacteria that improves intestinal barrier. *Akkermansia muciniphila* levels were shown to decrease after high fat diet [101].

Recently, different groups showed that bariatric surgery might induce weight loss not necessarily by a decrease in food intake and through malabsorption, but also by modulating the gut microbiota. Obese patients submitted to bariatric surgery experienced profound changes in the gut microbiota that correlated with weight loss, including: an increase in diversity, decrease in Firmicutes and methanogenic Archea, with concurrent increases in Bacterioidetes and Gammaproteobacteria, as well as a decrease in lactic acid bacteria such as *Lactobacillus* and *Bifidobacterium* [102,103,104]. Indeed, causality between modulation of gut microbiota and weight loss was proved by Liou *et al.* [105] Transfering the gut microbiota from mice that underwent bariatric surgery into non-operated germ-free mice, resulted in weight loss, decreased body and liver fat, as compared to germ-free mice receiving gut microbiota from mice submitted to sham surgery.

More recently, bile acids entered the equation between bariatric surgery, altered microbiota and weight loss. In fact, bariatric surgery is known to associate with increased circulating levels of bile acids and FXR signaling [106,107,108,109]. Suggesting a role of bile acids through FXR signaling, FXR deficient mice submitted to high-fat diet induced obesity and subsequent bariatric surgery (vertical sleeve gastrectomy), were less prone to sustained weight loss after surgery, with compensatory increase in food intake within three to five weeks [110]. Also, they did not improve glycemic control after surgery. Interestingly, as compared to wild type mice, in FXR deficient mice, bariatric surgery had an attenuated ability to modulate the gut microbiota, with no inhibition of Bacteroides and maintaining a decrease in *Roseburia* (known to also be decreased in human type 2 diabetes mellitus).

## 5. Microbiota as a Therapeutic Target

We can intervene in order to modulate our gut microbiota either giving commensal organisms known to improve our health status (dubbed probiotics), giving carbohydrates that stimulate the growth of potential beneficial commensals (dubbed prebiotics), or by giving a mix of both (dubbed symbiotics). In this review we will focus on the evidence on probiotics and symbiotics, since data on prebiotics alone are less robust.

Probiotics can potentially be beneficial in the treatment of NAFLD/NASH through several mechanisms: (a) competition with pathogenic species and antimicrobial effect modulating IgA secretion; (b) anti-inflammatory effect with inhibition of pro-inflammatory cytokines production; (c) increased gut satiety signals such as induction of YY peptide and inhibition of orexigenic ghrelin; (d) promotion of intestinal epithelium integrity and improvement of intestinal barrier; (e) decreased harvesting of energy from non-digestible carbohydrates; (f) decreased production of ethanol and other volatile organic compounds; (g) increased production of Fiaf; (h) decreased fatty acid oxidation in the liver; (i) insulin-sensitizing effect via synthesis of GLP-1; (j) modulation of bile acids and cholesterol metabolism; as well as (k) modulation of choline metabolism [13,111,112].

Due to the high resilience of our gut microbiota that easily tends to return to baseline after perturbation, interventions aimed to modulate the gut microbiota are deemed to early relapse to the initial dysbiota state after stopping the intervention, unless long-term approaches are used.

Several pre-clinical studies evaluated the role of probiotics in protecting from obesity and/or the metabolic syndrome, in different rodent models of obesity [113,114,115,116,117]. The studies are difficult to compare because not only are the models used different, the probiotics used are also different. While not all studies achieved a decrease in body weight and adiposity [117], all showed some metabolic benefit. Similarly, clinical studies in obese patients used different probiotics [118,119,120,121,122]. Those studies had small sample sizes and many of them were uncontrolled interventions [118,120,121]. Not all interventions achieved an improvement in body weight [118] or in metabolic profile [119,121]. While small pilot studies on prebiotics applied to obese patients did modify the gut microbiota [123] and improved lipid profile, in general those interventions failed to achieve weight loss or improvement in the glucose metabolism [124,125,126].

Probiotics have also been studied as a therapeutic tool for NAFLD/NASH. Three preclinical studies in mouse models of NAFLD associated with genetic and/or diet-induced obesity evaluated the role of a probiotic preparation, VSL#3. VSL#3 contains eight bacterial species from the genera *Bifidobacterium*, *Lactobacillus*, and *Streptococcus salivarius* subsp. thermophilus. This intervention improved steatosis, aminotransferases levels, serum lipids and insulin resistance [127,128,129]. Additionally, mice fed methionine-choline diet, a model of severe NASH not associated with obesity or the metabolic syndrome, developed less liver fibrosis when treated with VSL#3 [130]. Other probiotics also showed beneficial effects in animal models of NAFLD/NASH [131,132,133,134,135,136,137,138,139].

In humans, only small short-term pilot studies evaluated different probiotic/symbiotic preparations as a therapeutic approach for NAFLD (Table 2) [140,141,142,143,144,145,146,147]. However, the expectations on probiotics as a therapeutic tool in NAFLD are so high, that there are more systematic reviews and meta-analysis [13,112,148,149,150,151,152,153,154] on the topic than primary studies itself. Most studies did find a decrease in aminotransferases levels and hepatic steatosis after a short-term intervention. However, in terms of dysmetabolism, these studies failed to show benefit in anthropometric parameters and effect on lipid and glucose metabolism was not consistent among studies. Eslamparast *et al.* [146] noninvasively assessed liver fibrosis with transient elastography, pre- and post-intervention. They performed a randomized clinical trial, compared to placebo in 26 patients with NAFLD in each arm. They used a probiotic mixture that included different species from *Lactobacillus*, *Bifidobacterium*, and *Streptococcus* genera, as well as two different yeasts. After seven months of therapy, they did achieve a difference between probiotic and placebo arms in liver fibrosis, favoring the probiotic arm. One randomized clinical trial, with 36 patients with NASH in the probiotic group and 36 in the control group, performed liver biopsy pre and post-intervention [143]. After six months of treatment with Zirfos (a symbiotic with *B. longum*), patients in the symbiotic group, as compared to the placebo group, profited in terms of hepatic steatosis, but had no advantage in hepatocellular ballooning, liver inflammation, or liver fibrosis.

In summary, though promising, the evidence for the use of probiotics in the treatment of NAFLD/NASH is still insufficient. Studies are small, with short-term interventions, different formulations, different compositions of probiotics/symbiotics, and different durations of treatment. Also, most studies lack liver biopsy. The one study that systematically performed liver biopsy pre- and post-intervention failed to demonstrate significant differences between probiotics and placebo in important histological endpoints such as hepatic inflammation and fibrosis [143].

## 6. Conclusions

Obesity-associated NAFLD is the hepatic pandemic of our century. The gut microbiota has a huge impact in the pathogenesis of obesity and its metabolic complications, as well as in the development and progression of NAFLD. Gut dysbiosis promotes obesity through modulation of the energy harvested from the diet, as well as through direct modulation of adipose tissue and hepatic metabolism. Bacterial products may be toxic, two examples being ethanol and TMAO. Dysbiota may also perturb choline and bile acid metabolism, with detrimental effects in the liver. Furthermore, gut dysbiota can perturb the intestinal barrier, and bacterial products may induce systemic toxicity, including hepatic toxicity, that favors proinflammatory states and liver injury.

Several lines of evidence link NAFLD to dysbiosis; for example NAFLD associates with small bowel bacterial overgrowth, increased intestinal permeability, and endotoxemia. Also, in animal models of NAFLD/NASH, as well as in patients, the composition of the gut microbiota tends to be different from healthy subjects. Lastly, in animal models, NAFLD can be a transmissible disease by fecal microbiota transplantation from donors prone to develop NAFLD.

Taken into consideration the acknowledged role of gut dysbiosis in the pathogenesis of NAFLD/NASH, there are huge expectations on the role of probiotics/symbiotics in modulating the gut microbiota and hence having a therapeutic role in NAFLD. Despite the enthusiasm on the field, the available studies are small, heterogeneous, short-term, and do not properly address hepatic histology/risk for progressive liver disease. Hence, the lack of solid evidence, still precludes us implementing probiotics in the management of NAFLD/NASH. Extensive pre-clinical studies comparing different approaches in different animal models of NASH would be important to better delineate large multicentric well-designed, well-powered studies in patients with NASH. Other strategies for modulating the gut microbiota, such as fecal microbiota transplantation may merit further study.

## Figures and Tables

**Table 1:** Studies evaluating microbiota in human NAFLD/NASH.

Study	Population	Phyla	Class	Order	Family	Genera	Species
Spencer, M.D., 2011 [62]	15 women with a choline deficient diet and risk for NAFLD	Firmicutes	**↑ Erysipelotrichi**				
		Proteobacteriaceae	**↓ Gammaproteobacteria**				
Mouzaki, H., 2013 [63]	17 controls biopsy proven: 11 SS 22 NASH	**↓ Bacterioidetes**					
		Firmicutes	Clostridia	Clostridiales	Clostridiaceae	*Clostridium*	**↑ *C. coccoide***
Raman, H., 2013 [64]	30 obese with NAFLD 30 healthy controls	Firmicutes	Bacilli	Lactobacillales	Lactobacillaceae	**↑ *Lactobacillus***	
			Clostridia	Clostridiales	**↑ Lachnospiraceae**	**↑ *Robinsoniella*** **↑ *Roseburia*** **↑ *Dorea***	
					**↓ Ruminococceceae**	**↓ *Oscillibacter***	
Wong, V.W.S., 2013 [65]	16 NASH 22 healthy controls	Bacterioidetes	Bacterioidia	Bacteroidales	**↑ Porphyromonadaceae**	**↑ *Parabacteroides***	
		**↓ Firmicutes**	Clostridia	**↓ Clostridiales**	Clostridiaceae	**↓ *Faecalibacterium*** **↓ *Anaesporobacter***	
			Negativicutes	Selenomonodales	Veillonellaceae	**↑ *Allisonela***	
		Proteobacteria	Gammaproteobacteria	**↑ Aeromonadales**	**↑ Succinivibrionaceae**		
Boursier, J., 2015 [66]	57 patients with NAFLD: 30 F0/F1 27 > F1	Bacterioidetes	Bacterioidia	**↑ Bacterioidales** (NASH)			
		Firmicutes	Clostridia	Clostridiales	Rumminococceceae	**↑ Ruminococcus (>F1)**	
Jiang, W., 2015 [67]	53 NAFLD 32 healthy controls	Bacterioidetes	Bacterioidia	Bacteroidales	**↓ Porphyromonadaceae**	**↓ *Odoribacter***	
					Rikenellaceae	**↓ *Alistipes***	
					Prevotellaceae	**↓ *Prevotella***	
		Firmicutes	Bacilli	Lactobacillales	Lactobacillaceae	**↑ *Lactobacillus***	
					Streptococcaceae	**↑ *Streptococcus***	
			Clostridia	Clostridiales	Clostridiaceae	**↑ *Clostridium***	
					**↓ Ruminococceceae**	**↓ *Oscillibacter***	
						**↓ *Flavonitractor***	
		Proteobacteriaceae	Gammaproteobacteria	Enterobacteriales	Enterobactereaceae	**↑ *Escherichia***	
		**↓ Lentisphaerae**					

In bold are the associations described. NAFLD, nonalcoholic fatty liver disease; NASH, nonalcoholic steatohepatitis, F0/F1, no or mild fibrosis, >F1, significant fibrosis. Arrows indicate the differences in the studied group as compared to the control group.

**Table 2:** Studies evaluating the therapeutic role of probiotics in human NAFLD/NASH.

Study	Design	Probiotic Composition	Results
Loguercio, C., 2002 [140]	10 patients with NASH No control group Two months intervention	LAB: *L. acidophilus*, *L*, *rhamnosus*, *L plantarum*, *L. salivarius*, *L. casei*, *L. bulgaricus*, *B. lactis*, *B. bifidus*, *B. breve*, FOS, vitamins	↓ liver enzymes: ALT and γGT ↓ TNF-α levels and oxidative stress Relapse after stopping the intervention
Loguercio, C., 2005 [141]	22 patients with NASH No control group Three months intervention	VSL#3: *B. breve*, *B. longum*, *B. infantis*, *L. acidophilus*, *L. plantarum*, *L. paracasei*, *L. bulgaricus*, *S. Thermophilus* (2 capsules, twice a day)	↓ liver enzymes ↓ oxidative stress
Aller, R., 2011 [142]	Patients with NAFLD: probiotic group *n* = 15 and control group *n* = 15 Three months intervention	Mixture of 500 million *L. bulgaricus* + *S. thermophiles*	↓ liver enzymes: ALT no difference in anthropometric metrics no difference in lipid/glucose metabolism no difference in IL-6 or TNF-α levels
Malaguarnera, M., 2012 [143]	Patients with NASH: Probiotic group *n* = 34 and control group *n* = 29 Biopsy pre and post-intervention Six months intervention	Zirfos: FOS, *B. longum*, vitamins	↓ liver enzymes: AST ↓ LDL-cholesterol and insulin resistance ↓ TNF-α levels and endotoxemia no difference in anthropometric metrics ↓ steatosis and NAS score no difference in ballooning, inflammation or fibrosis
Wong, V.W.S., 2013 [144]	Patients with NASH: probiotic group *n* = 10 and control group *n* = 10 Six months intervention	Lepicol: *L. deslbrueckii*, *L. acidophilus*, *L. rhamnosus*, *B. bifidum*	↓ liver steatosis by H-MRS ↓ liver enzymes: AST no difference in anthropometric metrics no difference in lipid/glucose metabolism
Nabavi, S., 2014 [145]	Patients with NAFLD: probiotic group *n* = 36 and control group *n* = 36 Two months intervention	Probiotic yogurt containing *L. acidophilus* La5 and *B. lactis* Bb12	↓ liver enzymes: ALT and AST ↓ total cholesterol and LDL-cholesterol
Eslamparast, T., 2014 [146]	Patients with NAFLD: Probiotic group *n* = 26 and control group *n* = 26 Fibroscan© pre and post-intervention Seven months intervention	Protexin: *L. plantarum*, *L. bulgaricus*, *L. acidophilus*, *L. casei*, *B. bifidum*, *S. thermophilus*, *S. faecium*, *Torulopsis* spp, *Aspergillus oryzae*	↓ liver enzymes: AST, ALT and γGT ↓ TNF-α levels ↓ fibrosis assessed by transient elastography
Sepideh, A., 2015 [147]	Patients with NAFLD: probiotic group *n* = 21 and control group *n* = 21 Two months intervention	Lactocare: *L. casei*, *L. acidophilus*, *L. rhamnosus*, *L. bulgaricus*, *B. breve*, *B. longum*, *S. Thermophilus* (2 capsules per day)	↓ insulin resistance and IL-6 no difference in anthropometic metrics no difference in TNF-α levels

ALT, alanine aminotransferase; AST, aspartate aminotransferase; FOS, fructooligosaccharides; γGT, γ-glutamyl transpeptidase; H-MRS, proton magnetic resonance spectroscopy; IL-6, interleukin-6; LDL, low density lipoprotein; NAFLD, nonalcoholic fatty liver disease; NASH, nonalcoholic steatohepatitis, TNF-α, tumor necrosis factor α. Arrows indicate the differences in the intervention group as compared to the control group.

## References

[1] Bays H. (2014). Adiposopathy, “sick fat”, ockham’s razor, and resolution of the obesity paradox. Curr. Atheroscler. Rep..

[2] Byrne C.D., Targher G. (2015). NAFLD: A multisystem disease. J. Hepatol..

[3] Charlton M.R., Burns J.M., Pedersen R.A., Watt K.D., Heimbach J.K., Dierkhising R.A. (2011). Frequency and outcomes of liver transplantation for nonalcoholic steatohepatitis in the United States. Gastroenterology.

[4] Backhed F., Ding H., Wang T., Hooper L.V., Koh G.Y., Nagy A., Semenkovich C.F., Gordon J.I. (2004). The gut microbiota as an environmental factor that regulates fat storage. Proc. Natl. Acad. Sci. USA.

[5] Lagier J.C., Million M., Hugon P., Armougom F., Raoult D. (2012). Human gut microbiota: Repertoire and variations. Front. Cell. Infect. Microbiol..

[6] Gill S.R., Pop M., Deboy R.T., Eckburg P.B., Turnbaugh P.J., Samuel B.S., Gordon J.I., Relman D.A., Fraser-Liggett C.M., Nelson K.E. (2006). Metagenomic analysis of the human distal gut microbiome. Science.

[7] Seksik P., Landman C. (2015). Understanding microbiome data: A primer for clinicians. Dig. Dis..

[8] Lozupone C.A., Stombaugh J.I., Gordon J.I., Jansson J.K., Knight R. (2012). Diversity, stability and resilience of the human gut microbiota. Nature.

[9] Imajo K., Yoneda M., Ogawa Y., Wada K., Nakajima A. (2014). Microbiota and nonalcoholic steatohepatitis. Semin. Immunopathol..

[10] Martinez I., Muller C.E., Walter J. (2013). Long-term temporal analysis of the human fecal microbiota revealed a stable core of dominant bacterial species. PLoS ONE.

[11] Faith J.J., Guruge J.L., Charbonneau M., Subramanian S., Seedorf H., Goodman A.L., Clemente J.C., Knight R., Heath A.C., Leibel R.L. (2013). The long-term stability of the human gut microbiota. Science.

[12] Dethlefsen L., Relman D.A. (2011). Incomplete recovery and individualized responses of the human distal gut microbiota to repeated antibiotic perturbation. Proc. Natl. Acad. Sci. USA.

[13] Tarantino G., Finelli C. (2015). Systematic review on intervention with prebiotics/probiotics in patients with obesity-related nonalcoholic fatty liver disease. Future Microbiol..

[14] Donaldson G.P., Lee S.M., Mazmanian S.K. (2016). Gut biogeography of the bacterial microbiota. Nat. Rev. Microbiol..

[15] Backhed F., Manchester J.K., Semenkovich C.F., Gordon J.I. (2007). Mechanisms underlying the resistance to diet-induced obesity in germ-free mice. Proc. Natl. Acad. Sci. USA.

[16] Turnbaugh P.J., Ley R.E., Mahowald M.A., Magrini V., Mardis E.R., Gordon J.I. (2006). An obesity-associated gut microbiome with increased capacity for energy harvest. Nature.

[17] Turnbaugh P.J., Ridaura V.K., Faith J.J., Rey F.E., Knight R., Gordon J.I. (2009). The effect of diet on the human gut microbiome: A metagenomic analysis in humanized gnotobiotic mice. Sci. Transl. Med..

[18] Vrieze A., van Nood E., Holleman F., Salojarvi J., Kootte R.S., Bartelsman J.F., Dallinga-Thie G.M., Ackermans M.T., Serlie M.J., Oozeer R. (2012). Transfer of intestinal microbiota from lean donors increases insulin sensitivity in individuals with metabolic syndrome. Gastroenterology.

[19] Ley R.E., Backhed F., Turnbaugh P., Lozupone C.A., Knight R.D., Gordon J.I. (2005). Obesity alters gut microbial ecology. Proc. Natl. Acad. Sci. USA.

[20] Murphy E.F., Cotter P.D., Healy S., Marques T.M., O’Sullivan O., Fouhy F., Clarke S.F., O’Toole P.W., Quigley E.M., Stanton C. (2010). Composition and energy harvesting capacity of the gut microbiota: Relationship to diet, obesity and time in mouse models. Gut.

[21] Turnbaugh P.J., Backhed F., Fulton L., Gordon J.I. (2008). Diet-induced obesity is linked to marked but reversible alterations in the mouse distal gut microbiome. Cell Host Microbe.

[22] Turnbaugh P.J., Hamady M., Yatsunenko T., Cantarel B.L., Duncan A., Ley R.E., Sogin M.L., Jones W.J., Roe B.A., Affourtit J.P. (2009). A core gut microbiome in obese and lean twins. Nature.

[23] Patil D.P., Dhotre D.P., Chavan S.G., Sultan A., Jain D.S., Lanjekar V.B., Gangawani J., Shah P.S., Todkar J.S., Shah S. (2012). Molecular analysis of gut microbiota in obesity among indian individuals. J. Biosci..

[24] Ferrer M., Ruiz A., Lanza F., Haange S.B., Oberbach A., Till H., Bargiela R., Campoy C., Segura M.T., Richter M. (2013). Microbiota from the distal guts of lean and obese adolescents exhibit partial functional redundancy besides clear differences in community structure. Environ. Microbiol..

[25] Kalliomaki M., Collado M.C., Salminen S., Isolauri E. (2008). Early differences in fecal microbiota composition in children may predict overweight. Am. J. Clin. Nutr..

[26] Jumpertz R., Le D.S., Turnbaugh P.J., Trinidad C., Bogardus C., Gordon J.I., Krakoff J. (2011). Energy-balance studies reveal associations between gut microbes, caloric load, and nutrient absorption in humans. Am. J. Clin. Nutr..

[27] Chakraborti C.K. (2015). New-found link between microbiota and obesity. World J. Gastrointest. Pathophysiol..

[28] Brahe L.K., Astrup A., Larsen L.H. (2013). Is butyrate the link between diet, intestinal microbiota and obesity-related metabolic diseases?. Obes. Rev..

[29] Louis P., Flint H.J. (2009). Diversity, metabolism and microbial ecology of butyrate-producing bacteria from the human large intestine. FEMS Microbiol. Lett..

[30] Abdallah Ismail N., Ragab S.H., Abd Elbaky A., Shoeib A.R., Alhosary Y., Fekry D. (2011). Frequency of firmicutes and bacteroidetes in gut microbiota in obese and normal weight egyptian children and adults. Arch. Med. Sci..

[31] Roy C.C., Kien C.L., Bouthillier L., Levy E. (2006). Short-chain fatty acids: Ready for prime time?. Nutr. Clin. Pract..

[32] Lin H.V., Frassetto A., Kowalik E.J., Nawrocki A.R., Lu M.M., Kosinski J.R., Hubert J.A., Szeto D., Yao X., Forrest G. (2012). Butyrate and propionate protect against diet-induced obesity and regulate gut hormones via free fatty acid receptor 3-independent mechanisms. PLoS ONE.

[33] Xiong Y., Miyamoto N., Shibata K., Valasek M.A., Motoike T., Kedzierski R.M., Yanagisawa M. (2004). Short-chain fatty acids stimulate leptin production in adipocytes through the G protein-coupled receptor GPR41. Proc. Natl. Acad. Sci. USA.

[34] Al-Lahham S.H., Roelofsen H., Priebe M., Weening D., Dijkstra M., Hoek A., Rezaee F., Venema K., Vonk R.J. (2010). Regulation of adipokine production in human adipose tissue by propionic acid. Eur. J. Clin. Investig..

[35] Jin C.J., Sellmann C., Engstler A.J., Ziegenhardt D., Bergheim I. (2015). Supplementation of sodium butyrate protects mice from the development of non-alcoholic steatohepatitis (NASH). Br. J. Nutr..

[36] Aronsson L., Huang Y., Parini P., Korach-Andre M., Hakansson J., Gustafsson J.A., Pettersson S., Arulampalam V., Rafter J. (2010). Decreased fat storage by lactobacillus paracasei is associated with increased levels of angiopoietin-like 4 protein (ANGPTL4). PLoS ONE.

[37] Ding S., Chi M.M., Scull B.P., Rigby R., Schwerbrock N.M., Magness S., Jobin C., Lund P.K. (2010). High-fat diet: Bacteria interactions promote intestinal inflammation which precedes and correlates with obesity and insulin resistance in mouse. PLoS ONE.

[38] Claus S.P., Ellero S.L., Berger B., Krause L., Bruttin A., Molina J., Paris A., Want E.J., de Waziers I., Cloarec O. (2011). Colonization-induced host-gut microbial metabolic interaction. MBio.

[39] Rajala M.W., Patterson C.M., Opp J.S., Foltin S.K., Young V.B., Myers M.G. (2014). Leptin acts independently of food intake to modulate gut microbial composition in male mice. Endocrinology.

[40] Bergheim I., Weber S., Vos M., Kramer S., Volynets V., Kaserouni S., McClain C.J., Bischoff S.C. (2008). Antibiotics protect against fructose-induced hepatic lipid accumulation in mice: Role of endotoxin. J. Hepatol..

[41] Membrez M., Blancher F., Jaquet M., Bibiloni R., Cani P.D., Burcelin R.G., Corthesy I., Mace K., Chou C.J. (2008). Gut microbiota modulation with norfloxacin and ampicillin enhances glucose tolerance in mice. FASEB J..

[42] Drenick E.J., Fisler J., Johnson D. (1982). Hepatic steatosis after intestinal bypass—Prevention and reversal by metronidazole, irrespective of protein-calorie malnutrition. Gastroenterology.

[43] Nazim M., Stamp G., Hodgson H.J. (1989). Non-alcoholic steatohepatitis associated with small intestinal diverticulosis and bacterial overgrowth. Hepatogastroenterology.

[44] Wigg A.J., Roberts-Thomson I.C., Dymock R.B., McCarthy P.J., Grose R.H., Cummins A.G. (2001). The role of small intestinal bacterial overgrowth, intestinal permeability, endotoxaemia, and tumour necrosis factor α in the pathogenesis of non-alcoholic steatohepatitis. Gut.

[45] Brun P., Castagliuolo I., di Leo V., Buda A., Pinzani M., Palu G., Martines D. (2007). Increased intestinal permeability in obese mice: New evidence in the pathogenesis of nonalcoholic steatohepatitis. Am. J. Physiol. Gastrointest. Liver Physiol..

[46] Farhadi A., Gundlapalli S., Shaikh M., Frantzides C., Harrell L., Kwasny M.M., Keshavarzian A. (2008). Susceptibility to gut leakiness: A possible mechanism for endotoxaemia in non-alcoholic steatohepatitis. Liver Int..

[47] Spruss A., Kanuri G., Wagnerberger S., Haub S., Bischoff S.C., Bergheim I. (2009). Toll-like receptor 4 is involved in the development of fructose-induced hepatic steatosis in mice. Hepatology.

[48] Miele L., Valenza V., la Torre G., Montalto M., Cammarota G., Ricci R., Masciana R., Forgione A., Gabrieli M.L., Perotti G. (2009). Increased intestinal permeability and tight junction alterations in nonalcoholic fatty liver disease. Hepatology.

[49] Shanab A.A., Scully P., Crosbie O., Buckley M., O’Mahony L., Shanahan F., Gazareen S., Murphy E., Quigley E.M. (2011). Small intestinal bacterial overgrowth in nonalcoholic steatohepatitis: Association with toll-like receptor 4 expression and plasma levels of interleukin 8. Dig. Dis. Sci..

[50] Cani P.D., Amar J., Iglesias M.A., Poggi M., Knauf C., Bastelica D., Neyrinck A.M., Fava F., Tuohy K.M., Chabo C. (2007). Metabolic endotoxemia initiates obesity and insulin resistance. Diabetes.

[51] Verdam F.J., Rensen S.S., Driessen A., Greve J.W., Buurman W.A. (2011). Novel evidence for chronic exposure to endotoxin in human nonalcoholic steatohepatitis. J. Clin. Gastroenterol..

[52] Poggi M., Bastelica D., Gual P., Iglesias M.A., Gremeaux T., Knauf C., Peiretti F., Verdier M., Juhan-Vague I., Tanti J.F. (2007). C3H/HEJ mice carrying a toll-like receptor 4 mutation are protected against the development of insulin resistance in white adipose tissue in response to a high-fat diet. Diabetologia.

[53] Csak T., Velayudham A., Hritz I., Petrasek J., Levin I., Lippai D., Catalano D., Mandrekar P., Dolganiuc A., Kurt-Jones E. (2011). Deficiency in myeloid differentiation factor-2 and toll-like receptor 4 expression attenuates nonalcoholic steatohepatitis and fibrosis in mice. Am. J. Physiol. Gastrointest. Liver Physiol..

[54] Ye D., Li F.Y., Lam K.S., Li H., Jia W., Wang Y., Man K., Lo C.M., Li X., Xu A. (2012). Toll-like receptor-4 mediates obesity-induced non-alcoholic steatohepatitis through activation of X-box binding protein-1 in mice. Gut.

[55] Yuan L., Bambha K. (2015). Bile acid receptors and nonalcoholic fatty liver disease. World J. Hepatol..

[56] Stacey M., Webb M. (1947). Studies on the antibacterial properties of the bile acids and some compounds derived from cholanic acid. Proc. R. Soc. Med..

[57] Sayin S.I., Wahlstrom A., Felin J., Jantti S., Marschall H.U., Bamberg K., Angelin B., Hyotylainen T., Oresic M., Backhed F. (2013). Gut microbiota regulates bile acid metabolism by reducing the levels of tauro-β-muricholic acid, a naturally occurring fxr antagonist. Cell Metab..

[58] Le Roy T., Llopis M., Lepage P., Bruneau A., Rabot S., Bevilacqua C., Martin P., Philippe C., Walker F., Bado A. (2013). Intestinal microbiota determines development of non-alcoholic fatty liver disease in mice. Gut.

[59] Henao-Mejia J., Elinav E., Jin C., Hao L., Mehal W.Z., Strowig T., Thaiss C.A., Kau A.L., Eisenbarth S.C., Jurczak M.J. (2012). Inflammasome-mediated dysbiosis regulates progression of NAFLD and obesity. Nature.

[60] De Minicis S., Rychlicki C., Agostinelli L., Saccomanno S., Candelaresi C., Trozzi L., Mingarelli E., Facinelli B., Magi G., Palmieri C. (2014). Dysbiosis contributes to fibrogenesis in the course of chronic liver injury in mice. Hepatology.

[61] Yoshimoto S., Loo T.M., Atarashi K., Kanda H., Sato S., Oyadomari S., Iwakura Y., Oshima K., Morita H., Hattori M. (2013). Obesity-induced gut microbial metabolite promotes liver cancer through senescence secretome. Nature.

[62] Spencer M.D., Hamp T.J., Reid R.W., Fischer L.M., Zeisel S.H., Fodor A.A. (2011). Association between composition of the human gastrointestinal microbiome and development of fatty liver with choline deficiency. Gastroenterology.

[63] Mouzaki M., Comelli E.M., Arendt B.M., Bonengel J., Fung S.K., Fischer S.E., McGilvray I.D., Allard J.P. (2013). Intestinal microbiota in patients with nonalcoholic fatty liver disease. Hepatology.

[64] Raman M., Ahmed I., Gillevet P.M., Probert C.S., Ratcliffe N.M., Smith S., Greenwood R., Sikaroodi M., Lam V., Crotty P. (2013). Fecal microbiome and volatile organic compound metabolome in obese humans with nonalcoholic fatty liver disease. Clin. Gastroenterol. Hepatol..

[65] Wong V.W., Tse C.H., Lam T.T., Wong G.L., Chim A.M., Chu W.C., Yeung D.K., Law P.T., Kwan H.S., Yu J. (2013). Molecular characterization of the fecal microbiota in patients with nonalcoholic steatohepatitis—A longitudinal study. PLoS ONE.

[66] Boursier J., Mueller O., Barret M., Machado M., Fizanne L., Araujo-Perez F., Guy C.D., Seed P.C., Rawls J.F., David L.A. (2016). The severity of NAFLD is associated with gut dysbiosis and shift in the metabolic function of the gut microbiota. Hepatology.

[67] Jiang W., Wu N., Wang X., Chi Y., Zhang Y., Qiu X., Hu Y., Li J., Liu Y. (2015). Dysbiosis gut microbiota associated with inflammation and impaired mucosal immune function in intestine of humans with non-alcoholic fatty liver disease. Sci. Rep..

[68] Lebeer S., Vanderleyden J., de Keersmaecker S.C. (2008). Genes and molecules of lactobacilli supporting probiotic action. Microbiol. Mol. Biol. Rev..

[69] Elshaghabee F.M., Bockelmann W., Meske D., de Vrese M., Walte H.G., Schrezenmeir J., Heller K.J. (2016). Ethanol production by selected intestinal microorganisms and lactic acid bacteria growing under different nutritional conditions. Front. Microbiol..

[70] Scott K.P., Martin J.C., Duncan S.H., Flint H.J. (2014). Prebiotic stimulation of human colonic butyrate-producing bacteria and bifidobacteria, *in vitro*. FEMS Microbiol. Ecol..

[71] Prorok-Hamon M., Friswell M.K., Alswied A., Roberts C.L., Song F., Flanagan P.K., Knight P., Codling C., Marchesi J.R., Winstanley C. (2014). Colonic mucosa-associated diffusely adherent *afaC* + *Escherichia coli* expressing *lpfA* and *pks* are increased in inflammatory bowel disease and colon cancer. Gut.

[72] Al-Jashamy K., Murad A., Zeehaida M., Rohaini M., Hasnan J. (2010). Prevalence of colorectal cancer associated with streptococcus bovis among inflammatory bowel and chronic gastrointestinal tract disease patients. Asian Pac. J. Cancer Prev..

[73] Zhu L., Baker S.S., Gill C., Liu W., Alkhouri R., Baker R.D., Gill S.R. (2013). Characterization of gut microbiomes in nonalcoholic steatohepatitis (NASH) patients: A connection between endogenous alcohol and NASH. Hepatology.

[74] Zeisel S.H. (2010). Choline. Adv. Nutr..

[75] Dumas M.E., Barton R.H., Toye A., Cloarec O., Blancher C., Rothwell A., Fearnside J., Tatoud R., Blanc V., Lindon J.C. (2006). Metabolic profiling reveals a contribution of gut microbiota to fatty liver phenotype in insulin-resistant mice. Proc. Natl. Acad. Sci. USA.

[76] Ferreira D.M., Afonso M.B., Rodrigues P.M., Simao A.L., Pereira D.M., Borralho P.M., Rodrigues C.M., Castro R.E. (2014). c-jun N-terminal kinase 1/c-jun activation of the p53/microRNA 34a/sirtuin 1 pathway contributes to apoptosis induced by deoxycholic acid in rat liver. Mol. Cell. Biol..

[77] Aranha M.M., Cortez-Pinto H., Costa A., da Silva I.B., Camilo M.E., de Moura M.C., Rodrigues C.M. (2008). Bile acid levels are increased in the liver of patients with steatohepatitis. Eur. J. Gastroenterol. Hepatol..

[78] Newgard C.B. (2012). Interplay between lipids and branched-chain amino acids in development of insulin resistance. Cell Metab..

[79] Png C.W., Linden S.K., Gilshenan K.S., Zoetendal E.G., McSweeney C.S., Sly L.I., McGuckin M.A., Florin T.H. (2010). Mucolytic bacteria with increased prevalence in ibd mucosa augment *in vitro* utilization of mucin by other bacteria. Am. J. Gastroenterol..

[80] Sartor R.B. (2011). Key questions to guide a better understanding of host-commensal microbiota interactions in intestinal inflammation. Mucosal Immunol..

[81] Christopherson M.R., Dawson J.A., Stevenson D.M., Cunningham A.C., Bramhacharya S., Weimer P.J., Kendziorski C., Suen G. (2014). Unique aspects of fiber degradation by the ruminal ethanologen ruminococcus albus 7 revealed by physiological and transcriptomic analysis. BMC Genom..

[82] Yoneda M., Naka S., Nakano K., Wada K., Endo H., Mawatari H., Imajo K., Nomura R., Hokamura K., Ono M. (2012). Involvement of a periodontal pathogen, porphyromonas gingivalis on the pathogenesis of non-alcoholic fatty liver disease. BMC Gastroenterol..

[83] Furusho H., Miyauchi M., Hyogo H., Inubushi T., Ao M., Ouhara K., Hisatune J., Kurihara H., Sugai M., Hayes C.N. (2013). Dental infection of porphyromonas gingivalis exacerbates high fat diet-induced steatohepatitis in mice. J. Gastroenterol..

[84] Cope K., Risby T., Diehl A.M. (2000). Increased gastrointestinal ethanol production in obese mice: Implications for fatty liver disease pathogenesis. Gastroenterology.

[85] Nair S., Cope K., Risby T.H., Diehl A.M. (2001). Obesity and female gender increase breath ethanol concentration: Potential implications for the pathogenesis of nonalcoholic steatohepatitis. Am. J. Gastroenterol..

[86] Sajjad A., Mottershead M., Syn W.K., Jones R., Smith S., Nwokolo C.U. (2005). Ciprofloxacin suppresses bacterial overgrowth, increases fasting insulin but does not correct low acylated ghrelin concentration in non-alcoholic steatohepatitis. Aliment. Pharmacol. Ther..

[87] Swann J.R., Want E.J., Geier F.M., Spagou K., Wilson I.D., Sidaway J.E., Nicholson J.K., Holmes E. (2011). Systemic gut microbial modulation of bile acid metabolism in host tissue compartments. Proc. Natl. Acad. Sci. USA.

[88] Jiang C., Xie C., Li F., Zhang L., Nichols R.G., Krausz K.W., Cai J., Qi Y., Fang Z.Z., Takahashi S. (2015). Intestinal farnesoid X receptor signaling promotes nonalcoholic fatty liver disease. J. Clin. Investig..

[89] De Filippo C., Cavalieri D., di Paola M., Ramazzotti M., Poullet J.B., Massart S., Collini S., Pieraccini G., Lionetti P. (2010). Impact of diet in shaping gut microbiota revealed by a comparative study in children from europe and rural africa. Proc. Natl. Acad. Sci. USA.

[90] Walker A.W., Ince J., Duncan S.H., Webster L.M., Holtrop G., Ze X., Brown D., Stares M.D., Scott P., Bergerat A. (2011). Dominant and diet-responsive groups of bacteria within the human colonic microbiota. ISME J..

[91] David L.A., Maurice C.F., Carmody R.N., Gootenberg D.B., Button J.E., Wolfe B.E., Ling A.V., Devlin A.S., Varma Y., Fischbach M.A. (2014). Diet rapidly and reproducibly alters the human gut microbiome. Nature.

[92] Beyer P.L., Flynn M.A. (1978). Effects of high- and low-fiber diets on human feces. J. Am. Diet. Assoc..

[93] Cotillard A., Kennedy S.P., Kong L.C., Prifti E., Pons N., Le Chatelier E., Almeida M., Quinquis B., Levenez F., Galleron N. (2013). Dietary intervention impact on gut microbial gene richness. Nature.

[94] Wu G.D., Chen J., Hoffmann C., Bittinger K., Chen Y.Y., Keilbaugh S.A., Bewtra M., Knights D., Walters W.A., Knight R. (2011). Linking long-term dietary patterns with gut microbial enterotypes. Science.

[95] Hildebrandt M.A., Hoffmann C., Sherrill-Mix S.A., Keilbaugh S.A., Hamady M., Chen Y.Y., Knight R., Ahima R.S., Bushman F., Wu G.D. (2009). High-fat diet determines the composition of the murine gut microbiome independently of obesity. Gastroenterology.

[96] De Wit N., Derrien M., Bosch-Vermeulen H., Oosterink E., Keshtkar S., Duval C., de Vogel-van den Bosch J., Kleerebezem M., Muller M., van der Meer R. (2012). Saturated fat stimulates obesity and hepatic steatosis and affects gut microbiota composition by an enhanced overflow of dietary fat to the distal intestine. Am. J. Physiol. Gastrointest. Liver Physiol..

[97] Ravussin Y., Koren O., Spor A., LeDuc C., Gutman R., Stombaugh J., Knight R., Ley R.E., Leibel R.L. (2012). Responses of gut microbiota to diet composition and weight loss in lean and obese mice. Obesity.

[98] Brinkworth G.D., Noakes M., Clifton P.M., Bird A.R. (2009). Comparative effects of very low-carbohydrate, high-fat and high-carbohydrate, low-fat weight-loss diets on bowel habit and faecal short-chain fatty acids and bacterial populations. Br. J. Nutr..

[99] Fava F., Gitau R., Griffin B.A., Gibson G.R., Tuohy K.M., Lovegrove J.A. (2013). The type and quantity of dietary fat and carbohydrate alter faecal microbiome and short-chain fatty acid excretion in a metabolic syndrome “at-risk” population. Int. J. Obes..

[100] Zhang C., Zhang M., Wang S., Han R., Cao Y., Hua W., Mao Y., Zhang X., Pang X., Wei C. (2010). Interactions between gut microbiota, host genetics and diet relevant to development of metabolic syndromes in mice. ISME J..

[101] Everard A., Belzer C., Geurts L., Ouwerkerk J.P., Druart C., Bindels L.B., Guiot Y., Derrien M., Muccioli G.G., Delzenne N.M. (2013). Cross-talk between akkermansia muciniphila and intestinal epithelium controls diet-induced obesity. Proc. Natl. Acad. Sci. USA.

[102] Zhang H., DiBaise J.K., Zuccolo A., Kudrna D., Braidotti M., Yu Y., Parameswaran P., Crowell M.D., Wing R., Rittmann B.E. (2009). Human gut microbiota in obesity and after gastric bypass. Proc. Natl. Acad. Sci. USA.

[103] Furet J.P., Kong L.C., Tap J., Poitou C., Basdevant A., Bouillot J.L., Mariat D., Corthier G., Dore J., Henegar C. (2010). Differential adaptation of human gut microbiota to bariatric surgery-induced weight loss: Links with metabolic and low-grade inflammation markers. Diabetes.

[104] Kong L.C., Tap J., Aron-Wisnewsky J., Pelloux V., Basdevant A., Bouillot J.L., Zucker J.D., Dore J., Clement K. (2013). Gut microbiota after gastric bypass in human obesity: Increased richness and associations of bacterial genera with adipose tissue genes. Am. J. Clin. Nutr..

[105] Liou A.P., Paziuk M., Luevano J.M., Machineni S., Turnbaugh P.J., Kaplan L.M. (2013). Conserved shifts in the gut microbiota due to gastric bypass reduce host weight and adiposity. Sci. Transl. Med..

[106] Patti M.E., Houten S.M., Bianco A.C., Bernier R., Larsen P.R., Holst J.J., Badman M.K., Maratos-Flier E., Mun E.C., Pihlajamaki J. (2009). Serum bile acids are higher in humans with prior gastric bypass: Potential contribution to improved glucose and lipid metabolism. Obesity.

[107] Kohli R., Bradley D., Setchell K.D., Eagon J.C., Abumrad N., Klein S. (2013). Weight loss induced by Roux-en-Y gastric bypass but not laparoscopic adjustable gastric banding increases circulating bile acids. J. Clin. Endocrinol. Metab..

[108] Gerhard G.S., Styer A.M., Wood G.C., Roesch S.L., Petrick A.T., Gabrielsen J., Strodel W.E., Still C.D., Argyropoulos G. (2013). A role for fibroblast growth factor 19 and bile acids in diabetes remission after Roux-en-Y gastric bypass. Diabetes Care.

[109] Myronovych A., Kirby M., Ryan K.K., Zhang W., Jha P., Setchell K.D., Dexheimer P.J., Aronow B., Seeley R.J., Kohli R. (2014). Vertical sleeve gastrectomy reduces hepatic steatosis while increasing serum bile acids in a weight-loss-independent manner. Obesity.

[110] Ryan K.K., Tremaroli V., Clemmensen C., Kovatcheva-Datchary P., Myronovych A., Karns R., Wilson-Perez H.E., Sandoval D.A., Kohli R., Backhed F. (2014). Fxr is a molecular target for the effects of vertical sleeve gastrectomy. Nature.

[111] Shen W., Gaskins H.R., McIntosh M.K. (2014). Influence of dietary fat on intestinal microbes, inflammation, barrier function and metabolic outcomes. J. Nutr. Biochem..

[112] Ferolla S.M., Armiliato G.N., Couto C.A., Ferrari T.C. (2015). Probiotics as a complementary therapeutic approach in nonalcoholic fatty liver disease. World J. Hepatol..

[113] Kang J.H., Yun S.I., Park M.H., Park J.H., Jeong S.Y., Park H.O. (2013). Anti-obesity effect of lactobacillus gasseri BNR17 in high-sucrose diet-induced obese mice. PLoS ONE.

[114] Fak F., Backhed F. (2012). Lactobacillus reuteri prevents diet-induced obesity, but not atherosclerosis, in a strain dependent fashion in Apoe^−/−^ mice. PLoS ONE.

[115] Lee H.Y., Park J.H., Seok S.H., Baek M.W., Kim D.J., Lee K.E., Paek K.S., Lee Y., Park J.H. (2006). Human originated bacteria, lactobacillus rhamnosus pl60, produce conjugated linoleic acid and show anti-obesity effects in diet-induced obese mice. Biochim. Biophys. Acta.

[116] An H.M., Park S.Y., Lee do K., Kim J.R., Cha M.K., Lee S.W., Lim H.T., Kim K.J., Ha N.J. (2011). Antiobesity and lipid-lowering effects of *Bifidobacterium* spp. In high fat diet-induced obese rats. Lipids Health Dis..

[117] Andersson U., Branning C., Ahrne S., Molin G., Alenfall J., Onning G., Nyman M., Holm C. (2010). Probiotics lower plasma glucose in the high-fat fed C57BL/6J mouse. Benef. Microbes.

[118] Agerholm-Larsen L., Raben A., Haulrik N., Hansen A.S., Manders M., Astrup A. (2000). Effect of 8 week intake of probiotic milk products on risk factors for cardiovascular diseases. Eur. J. Clin. Nutr..

[119] Kadooka Y., Sato M., Imaizumi K., Ogawa A., Ikuyama K., Akai Y., Okano M., Kagoshima M., Tsuchida T. (2010). Regulation of abdominal adiposity by probiotics (*Lactobacillus gasseri* SBT2055) in adults with obese tendencies in a randomized controlled trial. Eur. J. Clin. Nutr..

[120] Mikirova N.A., Casciari J.J., Hunninghake R.E., Beezley M.M. (2011). Effect of weight reduction on cardiovascular risk factors and CD34-positive cells in circulation. Int. J. Med. Sci..

[121] Kadooka Y., Sato M., Ogawa A., Miyoshi M., Uenishi H., Ogawa H., Ikuyama K., Kagoshima M., Tsuchida T. (2013). Effect of *Lactobacillus gasseri* SBT2055 in fermented milk on abdominal adiposity in adults in a randomised controlled trial. Br. J. Nutr..

[122] Sanchez M., Darimont C., Drapeau V., Emady-Azar S., Lepage M., Rezzonico E., Ngom-Bru C., Berger B., Philippe L., Ammon-Zuffrey C. (2014). Effect of lactobacillus rhamnosus cgmcc1.3724 supplementation on weight loss and maintenance in obese men and women. Br. J. Nutr..

[123] Dewulf E.M., Cani P.D., Claus S.P., Fuentes S., Puylaert P.G., Neyrinck A.M., Bindels L.B., de Vos W.M., Gibson G.R., Thissen J.P. (2013). Insight into the prebiotic concept: Lessons from an exploratory, double blind intervention study with inulin-type fructans in obese women. Gut.

[124] de Luis D.A., de la Fuente B., Izaola O., Conde R., Gutierrez S., Morillo M., Teba Torres C. (2011). Double blind randomized clinical trial controlled by placebo with an α linoleic acid and prebiotic enriched cookie on risk cardiovascular factor in obese patients. Nutr. Hosp..

[125] De Luis D.A., de la Fuente B., Izaola O., Conde R., Gutierrez S., Morillo M., Teba Torres C. (2010). Randomized clinical trial with a inulin enriched cookie on risk cardiovascular factor in obese patients. Nutr. Hosp..

[126] Balcazar-Munoz B.R., Martinez-Abundis E., Gonzalez-Ortiz M. (2003). Effect of oral inulin administration on lipid profile and insulin sensitivity in subjects with obesity and dyslipidemia. Rev. Med. Chile.

[127] Li Z., Yang S., Lin H., Huang J., Watkins P.A., Moser A.B., Desimone C., Song X.Y., Diehl A.M. (2003). Probiotics and antibodies to TNF inhibit inflammatory activity and improve nonalcoholic fatty liver disease. Hepatology.

[128] Ma X., Hua J., Li Z. (2008). Probiotics improve high fat diet-induced hepatic steatosis and insulin resistance by increasing hepatic NKT cells. J. Hepatol..

[129] Esposito E., Iacono A., Bianco G., Autore G., Cuzzocrea S., Vajro P., Canani R.B., Calignano A., Raso G.M., Meli R. (2009). Probiotics reduce the inflammatory response induced by a high-fat diet in the liver of young rats. J. Nutr..

[130] Velayudham A., Dolganiuc A., Ellis M., Petrasek J., Kodys K., Mandrekar P., Szabo G. (2009). Vsl#3 probiotic treatment attenuates fibrosis without changes in steatohepatitis in a diet-induced nonalcoholic steatohepatitis model in mice. Hepatology.

[131] Xu R.Y., Wan Y.P., Fang Q.Y., Lu W., Cai W. (2012). Supplementation with probiotics modifies gut flora and attenuates liver fat accumulation in rat nonalcoholic fatty liver disease model. J. Clin. Biochem. Nutr..

[132] Bhathena J., Martoni C., Kulamarva A., Tomaro-Duchesneau C., Malhotra M., Paul A., Urbanska A.M., Prakash S. (2013). Oral probiotic microcapsule formulation ameliorates non-alcoholic fatty liver disease in Bio F1B Golden Syrian hamsters. PLoS ONE.

[133] Endo H., Niioka M., Kobayashi N., Tanaka M., Watanabe T. (2013). Butyrate-producing probiotics reduce nonalcoholic fatty liver disease progression in rats: New insight into the probiotics for the gut-liver axis. PLoS ONE.

[134] Ritze Y., Bardos G., Claus A., Ehrmann V., Bergheim I., Schwiertz A., Bischoff S.C. (2014). Lactobacillus rhamnosus GG protects against non-alcoholic fatty liver disease in mice. PLoS ONE.

[135] Xin J., Zeng D., Wang H., Ni X., Yi D., Pan K., Jing B. (2014). Preventing non-alcoholic fatty liver disease through lactobacillus johnsonii BS15 by attenuating inflammation and mitochondrial injury and improving gut environment in obese mice. Appl. Microbiol. Biotechnol..

[136] Li C., Nie S.P., Zhu K.X., Ding Q., Li C., Xiong T., Xie M.Y. (2014). Lactobacillus plantarum ncu116 improves liver function, oxidative stress and lipid metabolism in rats with high fat diet induced non-alcoholic fatty liver disease. Food Funct..

[137] Sohn W., Jun D.W., Lee K.N., Lee H.L., Lee O.Y., Choi H.S., Yoon B.C. (2015). Lactobacillus paracasei induces M2-Dominant Kupffer Cell Polarization in a Mouse Model of Nonalcoholic Steatohepatitis. Dig. Dis. Sci..

[138] Ting W.J., Kuo W.W., Hsieh D.J., Yeh Y.L., Day C.H., Chen Y.H., Chen R.J., Padma V.V., Chen Y.H., Huang C.Y. (2015). Heat killed lactobacillus reuteri GMNL-263 reduces fibrosis effects on the liver and heart in high fat diet-hamsters via TGF-β suppression. Int. J. Mol. Sci..

[139] Cortez-Pinto H., Borralho P., Machado J., Lopes M.T., Gato I.V., Santos A.M., Guerreiro A.S. (2016). Microbiota modulation with synbiotic decreases liver fibrosis in a high fat choline deficient diet mice model of nonalcoholic steatohepatitis (NASH). Port. J. Gastroenterol..

[140] Loguercio C., de Simone T., Federico A., Terracciano F., Tuccillo C., di Chicco M., Carteni M. (2002). Gut-liver axis: A new point of attack to treat chronic liver damage?. Am. J. Gastroenterol..

[141] Loguercio C., Federico A., Tuccillo C., Terracciano F., D’Auria M.V., de Simone C., del Vecchio Blanco C. (2005). Beneficial effects of a probiotic VSL#3 on parameters of liver dysfunction in chronic liver diseases. J. Clin. Gastroenterol..

[142] Aller R., de Luis D.A., Izaola O., Conde R., Gonzalez Sagrado M., Primo D., de la Fuente B., Gonzalez J. (2011). Effect of a probiotic on liver aminotransferases in nonalcoholic fatty liver disease patients: A double blind randomized clinical trial. Eur. Rev. Med. Pharmacol. Sci..

[143] Malaguarnera M., Vacante M., Antic T., Giordano M., Chisari G., Acquaviva R., Mastrojeni S., Malaguarnera G., Mistretta A., Li Volti G. (2012). Bifidobacterium longum with fructo-oligosaccharides in patients with non alcoholic steatohepatitis. Dig. Dis. Sci..

[144] Wong V.W., Won G.L., Chim A.M., Chu W.C., Yeung D.K., Li K.C., Chan H.L. (2013). Treatment of nonalcoholic steatohepatitis with probiotics. A proof-of-concept study. Ann. Hepatol..

[145] Nabavi S., Rafraf M., Somi M.H., Homayouni-Rad A., Asghari-Jafarabadi M. (2014). Effects of probiotic yogurt consumption on metabolic factors in individuals with nonalcoholic fatty liver disease. J. Dairy Sci..

[146] Eslamparast T., Poustchi H., Zamani F., Sharafkhah M., Malekzadeh R., Hekmatdoost A. (2014). Synbiotic supplementation in nonalcoholic fatty liver disease: A randomized, double-blind, placebo-controlled pilot study. Am. J. Clin. Nutr..

[147] Sepideh A., Karim P., Hossein A., Leila R., Hamdollah M., Mohammad E.G., Mojtaba S., Mohammad S., Ghader G., Seyed Moayed A. (2015). Effects of multistrain probiotic supplementation on glycemic and inflammatory indices in patients with nonalcoholic fatty liver disease: A double-blind randomized clinical trial. J. Am. Coll.Nutr..

[148] Lirussi F., Mastropasqua E., Orando S., Orlando R. (2007). Probiotics for non-alcoholic fatty liver disease and/or steatohepatitis. Cochrane Database Syst. Rev..

[149] Abenavoli L., Scarpellini E., Rouabhia S., Balsano C., Luzza F. (2013). Probiotics in non-alcoholic fatty liver disease: Which and when. Ann. Hepatol..

[150] Kelishadi R., Farajian S., Mirlohi M. (2013). Probiotics as a novel treatment for non-alcoholic fatty liver disease; a systematic review on the current evidences. Hepat. Mon..

[151] Ma Y.Y., Li L., Yu C.H., Shen Z., Chen L.H., Li Y.M. (2013). Effects of probiotics on nonalcoholic fatty liver disease: A meta-analysis. World J. Gastroenterol..

[152] Eslamparast T., Eghtesad S., Hekmatdoost A., Poustchi H. (2013). Probiotics and nonalcoholic fatty liver disease. Middle East J. Dig. Dis..

[153] Buss C., Valle-Tovo C., Miozzo S., Alves de Mattos A. (2014). Probiotics and synbiotics may improve liver aminotransferases levels in non-alcoholic fatty liver disease patients. Ann. Hepatol..

[154] Gao X., Zhu Y., Wen Y., Liu G., Wan C. (2016). Efficacy of probiotics in nonalcoholic fatty liver disease in adult and children: A meta-analysis of randomized controlled trials. Hepatol. Res..

